# Identifying Drug Sensitivity Subnetworks with NETPHIX

**DOI:** 10.1016/j.isci.2020.101619

**Published:** 2020-09-29

**Authors:** Yoo-Ah Kim, Rebecca Sarto Basso, Damian Wojtowicz, Amanda S. Liu, Dorit S. Hochbaum, Fabio Vandin, Teresa M. Przytycka

**Affiliations:** 1 National Center of Biotechnology Information, National Library of Medicine, NIH, Bethesda, MD 20894, USA; 2 Department of Industrial Engineering and Operations Research, University of California at Berkeley, Berkeley, CA 94709, USA; 3 Montgomery Blair High School, Silver Spring, MD 20901, USA; 4 Department of Information Engineering, University of Padova, Padova 35131, Italy

**Keywords:** Biological Sciences, Bioinformatics, Cancer Systems Biology

## Abstract

Phenotypic heterogeneity in cancer is often caused by different patterns of genetic alterations. Understanding such phenotype-genotype relationships is fundamental for the advance of personalized medicine. We develop a computational method, named NETPHIX (NETwork-to-PHenotype association with eXclusivity) to identify subnetworks of genes whose genetic alterations are associated with drug response or other continuous cancer phenotypes. Leveraging interaction information among genes and properties of cancer mutations such as mutual exclusivity, we formulate the problem as an integer linear program and solve it optimally to obtain a subnetwork of associated genes. Applied to a large-scale drug screening dataset, NETPHIX uncovered gene modules significantly associated with drug responses. Utilizing interaction information, NETPHIX modules are functionally coherent and can thus provide important insights into drug action. In addition, we show that modules identified by NETPHIX together with their association patterns can be leveraged to suggest drug combinations.

## Highlights

• NETPHIX identifies mutated subnetworks associated with a continuous phenotype
• It can aid the identification of mutated modules associated with drug response
• NETPHIX modules can be used to predict drug response and infer drug combinations
• NETPHIX uncovers subnetworks using Integer Linear Programming optimization

## Graphical Abstract

**Figure:**
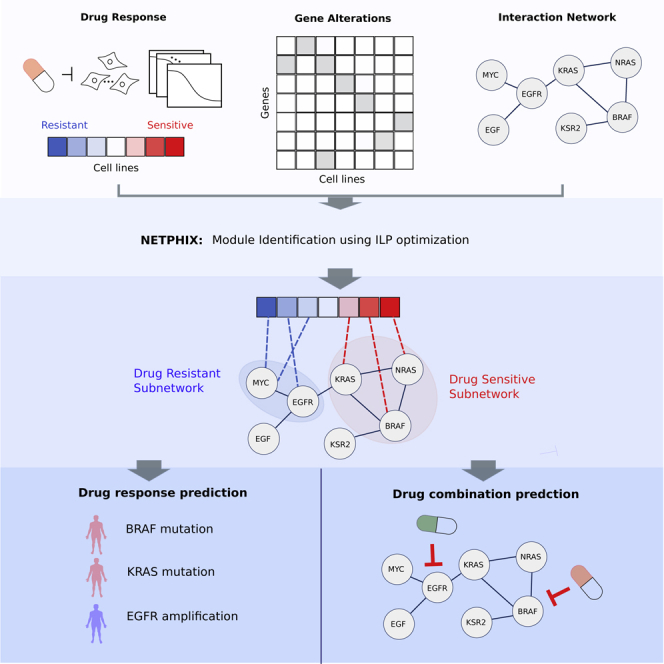

## Introduction

Genetic alterations in cancer are associated with diverse phenotypic properties such as drug response or patient survival. However, the identification of mutations causing specific phenotypes and the interpretation of the phenotype-genotype relationships remain challenging owing to a large number of passenger mutations and cancer heterogeneity. Indeed, the relationships between genotype and phenotype in most tumors are complex and different mutations in functionally related genes can lead to the same phenotype.

Predicting drug responses and identifying their genotypic causes is one of the most critical problems in cancer studies and crucial for developing personalized treatments. Pharmaceutical drugs are often developed to target specific genes, and the response depends on the function and the mutation status of the gene as well as other genes in the same or related pathways. Recently, several projects have characterized drug sensitivity in hundreds of cancer cell lines for a large number of drugs (Yang et al., 2013; Barretina et al., 2012). These data, together with information about the genetic alterations in these cell lines, provided unprecedented opportunities to understand how genetic alterations affect drug sensitivity.

The pathway-centric view of cancer (Hanahan and Weinberg 2011; Garraway and Lander 2013; Vogelstein et al., 2013) suggests that cancer phenotype should be considered from the context of dysregulated pathways rather than from the perspective of mutations in individual genes. Such a pathway-centric view significantly advanced the understanding of the mechanisms of tumorigenesis. Many computational methods to identify cancer driving mutations have been developed based on pathway-centric approaches (Chuang et al., 2007; Vandin et al., 2012; Kim et al., 2013; Hofree et al., 2013; Kim et al., 2016a; Dao et al., 2017). Network-based approaches have been further extended to find genes whose mutations are associated specifically with given phenotypes rather than finding general cancer drivers (Gilman et al., 2012; Hofree et al., 2013; Carter et al., 2013; Kim et al., 2016a; Zhang et al., 2018). Although the success of network-based methods in other cancer domains suggests that such approaches should be also useful in the studies of drug response, most of the aforementioned network-based approaches focused on identifying mutations associated with discrete phenotypic traits, e.g., cancer versus healthy, good or bad prognosis, or cancer subtypes, and, therefore, cannot be directly applied to the analysis of continuous features such as drug sensitivity.

There have been several methods proposed for predicting drug response in cancer, including a few network-based approaches (Emad et al., 2017; Han et al., 2019; Wang et al., 2019; Ali and Aittokallio, 2019; Chen and Zhang, 2020). For example, ProGENI adopts a random walk approach on a gene interaction network to rank genes based on their association with drug response and their expression status, subsequently using highly ranked genes for drug response prediction Emad et al. (2017). However, ProGENI and most of the algorithms cited above focus on response prediction, primarily relying on transcriptomic information rather than identifying mutational biomarkers associated with drug responses. Computational methods that make a better use of CNV and point mutation data considering their unique properties are still in need of development (Ali and Aittokallio, 2019).

Another body of related studies is combining GWAS analysis with network constraints (Li and Li, 2008; Jia et al., 2011; Azencott et al., 2013; Liu et al., 2017). Although these methods generally perform well at broadly pointing to disease-related genes, they do not consider complex properties of cancer mutations such as frequently observed mutual exclusivity of cancer mutations, and are not designed to zoom in on subnetworks that are specific enough to help understand drug action.

Several algorithms have considered a problem closely related to our work—identifying mutations associated with drug response—but without including functional relationship information (Kim et al., 2016; Sarto Basso et al., 2019; Knijnenburg et al., 2016). For example, REVEALER used a re-scaled mutual information metric to iteratively identify a set of genes associated with a phenotype Kim et al. (2016). UNCOVER employs an integer linear programming formulation based on the set cover problem, by designing the objective function to maximize the association with the phenotype and preferentially select mutually exclusive gene sets Sarto Basso et al. (2019). Although UNCOVER uses a similar objective function as NETPHIX (NETwork-to-PHenotype assocIation with eXclusivity), it does not utilize network information nor allows to pick up mixed sensitivity modules (i.e., simultaneously identify genes associated with increased and decreased drug sensitivity). LOBICO (Knijnenburg et al., 2016; Iorio et al., 2016) is another algorithm designed to identify a set of genes whose alterations are associated with differences in drug response. The algorithm is formulated as an integer linear program, based on logic models of binary input features that explain a continuous phenotype variable. However, none of the algorithms mentioned above utilizes network interaction information. Since perturbations in functionally related genes are likely to lead to similar phenotypes, functional interaction information can be helpful for the identification of phenotype-associated genes.

Having the network-centric views in mind, we introduce a computational tool named for identifying mutated subnetworks that are associated with a continuous phenotype. Our algorithm builds on combinatorial optimization techniques involving *connected set cover* to find a connected set of genes associated with increased or decreased sensitivity. The objective function of NETPHIX allows different options to capture various properties of cancer mutations: First, both drug-resistant and -sensitive genes can be identified simultaneously, considering interactions between them. Second, by having an option to impose penalties for overlapping mutations, the algorithm can preferentially select mutually exclusive genes in the solution. Based on the observation that cancer-related mutations tend to be mutually exclusive (Ciriello et al., 2013; Kim et al., 2015, 2016b; Vandin et al., 2012; Leiserson et al., 2015; Constantinescu et al., 2015), we hypothesized that mutual exclusivity may also be useful for the identification of drug sensitivity modules.

We evaluated NETPHIX and other related methods using both simulations and real drug screening datasets and showed that NETPHIX outperforms the competing methods. Applying NETPHIX to a large-scale drug response data (Genomics of Drug Sensitivity in Cancer [GDSC]), we identified sensitivity-associated subnetworks for many of the drugs, which provided important insights into drug action. We were also able to validate many of the identified modules with an independent drug screening dataset (The Cancer Therapeutics Response Portal [CTRP]). Finally, we show that properties of modules associated with drug can point to potential drug combinations. Effective computational methods to discover genetic alterations causal to drug sensitivity will improve our understanding of the molecular mechanism of drug sensitivity, help to identify potential drug combinations, and have a profound impact on genome-driven, personalized drug therapy.

Besides drug sensitivity profiles, NETPHIX can be applied to other continuous phenotypes. For instance, a simplified version of our method was used to investigate the genetic aberrations associated with mutational signatures, providing novel insights into mutagenic processes that the cancer genomes might have undergone Kim et al. (2020). Furthermore, the mutual exclusivity condition can be easily removed and therefore NETPHIX can also be used for the more general problem of linking continuous phenotypes (unrelated to cancer) to genetic alterations.

## Results

### NETPHIX Method Overview

**Figure 1:**
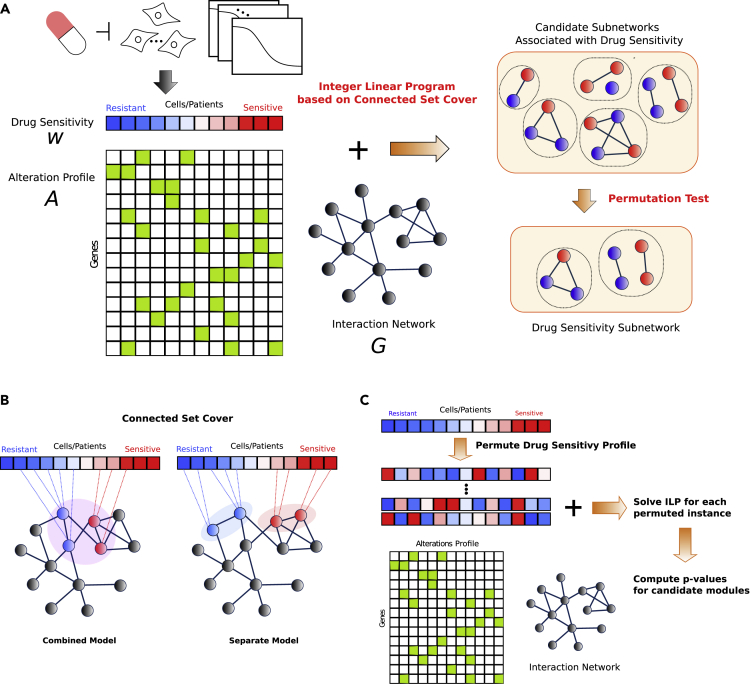
NETPHIX Method (A) Overview: NETPHIX takes drug sensitivity profile, alterations status for the same set of samples, and interaction information among genes as inputs. Using a connected set cover-based ILP algorithm, we first generate a set of candidate modules. The final set of modules include only maximal modules among statistically significant solutions. Genes associated with decreased and increased sensitivity are marked as blue and red, respectively. (B) NETPHIX finds a connected set of genes of which alterations are associated with phenotype values (red colors in the drug response profile indicate increased sensitivity values and blue colors are for decreased sensitivity values). We considered the *combined* model in which all the selected genes are connected and the *separate* model in which two subnetworks are identified for increased and decreased sensitivity separately. (C) The significance of identified modules is assessed using a permutation test by permuting drug sensitivity profiles.

NETPHIX takes gene alteration information, drug response profiles, and interaction network as inputs and identifies genetic alterations underlying the phenotype of interest (Figure 1A). In the first phase, we generate candidate modules solving an optimization problem based on a connected set cover approach as described in section Connected Set Cover Based Algorithm for Selecting Candidate Modules. The problem is formulated as an integer linear program (ILP), and candidate modules are generated by obtaining the optimal solutions using CPLEX (https://www.ibm.com/analytics/cplex-optimizer) for ILP instances with different parameters. In the second phase, the statistical significance of candidate modules is assessed with a permutation test, and final sensitivity modules are selected (see sections Selecting Final Modules and S1.1.4 for details).

#### Connected Set Cover-Based Algorithm for Selecting Candidate Modules

To obtain candidate subnetworks, we design our algorithm based on connected set cover to maximize the association with drug response (Figure 1B). Connected set cover approaches have been used successfully for the identification of cancer driving mutations, to overcome the challenges posed by the heterogeneity of cancer mutations and to help uncover relevant genes with rare or medium mutation frequencies (Kim et al., 2013, 2015; Sarto Basso et al., 2019; Kim et al., 2011; Chowdhury and Koyuturk, 2010; Ulitsky et al., 2010; Hristov and Singh, 2017). Below we describe how the technique can be extended to identify modules associated with drug response. For the formal definition of the problem, see Section S1.1.1.

NETPHIX takes a gene alteration matrix *A* and drug response profile *w* for a set of patients *P* (or cell lines) as inputs. Alteration matrix *A* is a |V|×|P| binary matrix where A(v,p)=1 if gene *v* is altered in patient p and 0 otherwise. *w* is a vector of length |P| (Figure 1A). In addition, an interaction network G=(V,E) is given where *V* represents genes. NETPHIX then aims to identify a set of connected genes S⊆V so as to maximize

(Equation 1) $$W\left(S\right)=\underset{p\in P\left(S\right)}{\sum }w\left(p\right)$$

where P(S) is the set of patients who have alterations in any genes in *S*. In other words, the algorithm tries to *cover* drug-sensitive patients with genes having alterations in the patients while maximizing the total weight of covered patients and enforcing the identified genes to be *connected* in the network. The connected set cover approach can capture the heterogeneity of cancer mutations as different patients who are sensitive to a drug may have causal alterations in (or are covered by) different but functionally related genes.

In addition, the objective function of NETPHIX can include penalties for overlapping mutations in covered patients to reinforce the property of mutual exclusivity in the selected modules. Based on the observation that cancer-related mutations tend to be mutually exclusive, we hypothesized that the property may also be useful for the identification of drug sensitivity modules. To include penalties for overlapping mutations, we can extend the objective function as follows:

(Equation 2) $$W\left(S\right)=\underset{p\in P\left(S\right)}{\sum }w\left(p\right)-\underset{p\in P\left(S\right)}{\sum }pt\left(p\right)\left(c\left(p,S\right)-1\right)$$

where c(p,S)=|{g∈S|A(g,p)=1}| and pt(p) is the penalty for overlapping mutations in patient *p*.

Although it is natural to assume that genes associated with a specific response are likely from a functional subnetwork, it was not clear what relation should be assumed between modules with association in opposite directions. Therefore, we considered two different connectivity models (Figure 1B) as there can be genes associated with either direction of drug response—genes whose alterations correlate with increased sensitivity to the drug (sensitive) and genes whose alterations correlate with decreased sensitivity to the drug (resistant). NETPHIX finds a module that includes both types of genes simultaneously using two different models—the combined model and the separate model. In the “combined” model, we identify one connected subnetwork that includes all genes associated with either direction (decreased or increased drug sensitivity). In the “separate” model, we seek to identify two subnetworks, one for increased sensitivity and one for decreased sensitivity separately. This model is designed to capture the case when two different functional modules affect drug response in different ways.

We create multiple ILP instances for different module sizes (i.e., the number of genes in the module) and connectivity options and obtain candidate modules by solving the ILP instances optimally with CPLEX. For the detailed ILP formulation and parameters, see Sections S1.1.2 and S1.1.3.

#### Selecting Final Modules

Once we obtain the optimal gene module for each parameter combination, the significance of the identified module is assessed by performing a permutation test (Figure 1C). Note that our algorithm is designed to identify the modules associated specifically with a given phenotype (e.g., drug sensitivity to each drug) rather than finding general cancer drivers. Therefore, a permutation test was performed by permuting the drug sensitivity profile so that the significance of the association is assessed in comparison with randomly generated phenotypes. Among all significantly associated subnetworks, we obtain the final drug sensitivity modules by selecting maximal modules to remove redundancy. In other words, for any two significant modules $M_{i}$ and $M_{j}$ such that $M_{i}\subset M_{j}$, only $M_{j}$ is included in the final solution for the drug. See Section S1.1.4 for the details of the permutation test and maximal module selection.

### Method Evaluation

#### Evaluation with Simulation Data

We first compared the performance of NETPHIX using simulation data with four other related methods—LOBICO, UNCOVER, SigMOD, and ProGENI. Of the four algorithms, SigMOD and ProGENI are network-based algorithms. ProGENI adopts a random walk approach in an interaction network and ranks genes based on their associations between drug response and gene aberrations Emad et al. (2017). SigMOD is a recently proposed module identification algorithm combining GWAS and a network-based approach Liu et al. (2017). SigMOD requires individual association scores of genes to a phenotype as an input, for which we used the p value of association of each gene to a given phenotype by performing t tests on the coefficients of univariate linear regression. LOBICO is a logic model-based algorithm, developed to identify a set of genes whose alterations are related to drug response Knijnenburg et al. (2016). UNCOVER Sarto Basso et al. (2019) was proposed as a method to identify a set of phenotype-associated genes by taking a set cover approach similar to ours. Although both LOBICO and UNCOVER find an optimal solution using an integer linear program, neither algorithms utilize interaction network information.

**Figure 2:**
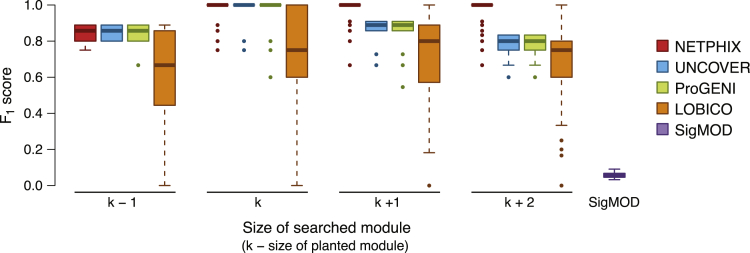
Method Comparison on Simulated Data $F_{1}$ score for the modules identified by NETPHIX (red), UNCOVER (blue), ProGENI (green), LOBICO (orange), and SigMOD (purple).

The simulated instances are generated by randomly generating drug response profiles and planting a connected set of genes so that their alteration patterns are associated with the drug response. We generated the instances with varying parameters and planted on the background of real cancer cell line mutation data and the functional interaction network information downloaded from STRING database (https://string-db.org) (see Section S1.3.1 for the detailed description). Although NETPHIX can identify subnetworks with mixed associations simultaneously, UNCOVER considers each direction separately. In addition, the logic models of LOBICO become more complicated and difficult to solve when both sides of associations are present. Therefore, in order not to disadvantage any of the methods, for the simulation-based evaluation we considered simple cases where alterations are associated with only one direction (either increased or decreased). We planted modules of sizes 3, 4, and 5 (the module size refers to the number of genes in the selected module) and evaluated the accuracy of the five methods in identifying the planted modules in terms of F1 scores (Figure 2; see Figures S1A and S1B for precision and recall). For all algorithms except SigMOD and ProGENI, we ran the algorithm for different $k^{\prime }$s ($k^{\prime }$ is a parameter for the size of a module searched by the algorithms). Since ProGENI ranks genes instead of selecting modules, we considered the top $k^{\prime }$ genes to be the selected modules. SigMOD automatically adjusts its parameters to find the best module. Also, for all ILP-based algorithm, we limit the running time up to 24 h, meaning the algorithms will stop and output the current solution (which may be suboptimal) when the time limit reaches.

As shown in Figure 2, NETPHIX outperforms other algorithms, especially when the algorithms search for bigger modules than planted. Note that, in real applications, the size of a module is unknown; therefore, the ability of correctly estimating the size of the module is an important component of a method. Since the genes should be connected, NETPHIX usually does not extend the best module with spurious genes even if we search for modules bigger than planted. On the other hand, other algorithms tend to add more genes when increasing $k^{\prime }$. In general, in this basic scenario, the algorithms uncovered the planted modules in most instances (Figure S1B) as long as the size of searched modules are at least as big as the planted module sizes, but LOBICO solutions missed true positives more often compared with other algorithms. SigMOD identified a large number of false positives along with the planted modules (approximately 100–180 genes) that are not associated with phenotypes.

#### Evaluation with Real Drug Screening Dataset

We next evaluated the performance of algorithms with real drug screening dataset (GDSC) and further validated the results on an independent set (CTRP). We included ProGENI and UNCOVER for comparison since the performance of these methods on simulated data was fairly good.

In the GDSC dataset, the responses for 265 drugs are available for 240–705 cell lines depending on drugs. For the alteration table, 26,917 gene-level alteration profiles are collected, combining amplification, deletion, and somatic mutations (see Section S1.2 for a detailed description of the data, including criteria used to identify genomic alterations).

NETPHIX identified a total of 476 modules for 194 drugs (for the remaining drugs no modules with significant association were identified, Table S1). Since there can be multiple functional modules affecting drug efficacy, our method allows us to identify multiple associated modules for a specific drug. Of 476 identified modules, 258 modules consist of one connected module based on the combined model (for 163 drugs) and 218 modules consist of two connected components based on the separate model (for 136 drugs). UNCOVER identified modules for 127 drugs (p value < 0.05) and only those modules were tested; 114 drugs had both NETPHIX and UNCOVER modules identified. ProGENI ranks genes and does not provide significance scores, and therefore, top ranked genes for all 265 drugs were used for comparison.

We compared the performance in terms of several measures. First, the distances between the modules and the drug targets were computed to examine the relationship between them. Next, the predictive power of drug responses for the identified genes were measured using probabilistic concordance index (PCI). In addition, we computed p values with ANOVA test to examine if the alteration status of the selected genes is associated with drug response.

##### Distance to Drug Targets

**Figure 3:**
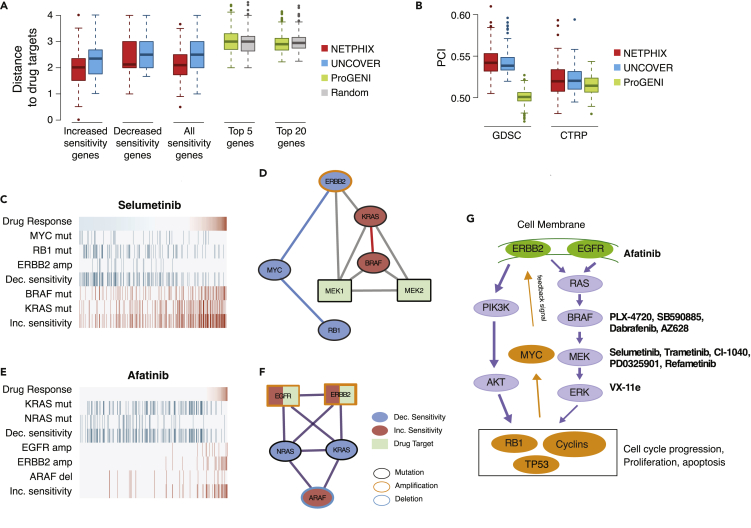
Evaluation Results and Modules Identified with Real Drug Screening Dataset (A–B) Evaluation with drug screening dataset. (A) Average distances of selected genes to drug targets. Distances for genes associated with increased sensitivity, decreased sensitivity, all genes in the selected modules are shown for NETPHIX and UNCOVER. The distances with 5 and 20 top ranked genes for ProGENI and randomly selected genes are also shown. (B) Predictive power of the modules selected by NETPHIX, UNCOVER, and ProGENI. PCI scores with random forest regression tested within GDSC and with CTRP dataset are shown. (C–F) Sensitivity networks identified by NETPHIX. Alternation profile and connectivity between the drug target and the genes for Selumetinib (C, D) and Afatinib (E, F). In the alteration profile, the panel shows the values of the phenotype (i.e., drug response, top row) for all samples (columns), with blue being decreased sensitivity values and red being increased sensitivity values. For each gene, alteration status in each sample is shown in red/blue (with the summary covers for decreased and increased sensitivity separately), whereas samples not altered are shown in gray. The module for Selumetinib is identified based on the separate connectivity model, and the module for Afatinib is selected based on the combined connectivity model. (G) Schematic diagram of MAPK/ERK and AKT signaling pathways with drugs and their drug targets annotated.

We first examined the relationship between the identified modules and drug targets. Specifically, given that we expect a mutated module to be related to the action of a drug, the module is likely to be close to the drug target(s) in the network. To test this, we computed the distances between the drug targets and the genes selected by each method for the corresponding drugs (Figure 3A). In case of NETPHIX the genes in drug sensitivity modules are located close to the corresponding drug targets in the network (the mean distances of 2.12, whereas the average distance to targets for randomly selected set of genes is greater than 2.97; $p<10^{-57}$, t test). For UNCOVER, the distances are larger than for NETPHIX (the mean distances of 2.5) but still smaller than random ($p<10^{-11}$). ProGENI genes have similar statistics as random genes.

As NETPHIX selects genes associated with both decreased and increased sensitivity, we also examined if there are differences between the two sets. Interestingly, we observed that the genes associated with increased sensitivity are closer to drug targets than the genes associated with decreased sensitivity (the mean distance 1.97 versus 2.37, $p<10^{-13}$, t test), indicating that the efficacy of a drug improves if it targets genes that are close to perturbed gene modules. We observed similar patterns in UNCOVER modules albeit less significant (the mean distance 2.23 versus 2.67, $p<10^{-3}$).

##### Predictive Power of Selected Modules Using Regression

Next, we examined the ability of predicting drug responses for the modules identified by different algorithms. To this purpose, we performed random forest (RF) regression using the selected genes as features. As NETPHIX selects multiple modules for some drugs, we merged all modules significantly associated with the response for each drug and used all selected genes as features for RF regression. The Boolean indicators of mutation status were used as feature values. The number of features for each drug varies between 3 and 15 in total. UNCOVER finds at most one significant module for decreased and increased sensitivity, respectively, and we used genes in both directions as features (total of 3–6 genes). For ProGENI, we used 20 top ranked genes as features for regression. We first performed a nested cross-validation on GDSC dataset by learning the best model with training set and testing with the remaining set of cell lines within GDSC (see Section S1.3.2 for details). In the second set of evaluation, we learned hyperparameters from GDSC dataset and applied the best model to an independent dataset (CTRP) and examined the predictive power. As shown in Figure 3B, the predictive power of NETPHIX outperformed other algorithms in both measurements, although UNCOVER had comparable prediction results for CTRP.

##### Predictive Power of Selected Modules Using ANOVA Test

For another way to examine if the alterations in the identified modules indeed lead to specific responses for the corresponding drugs, we divided cell lines into different groups depending on their alteration status and tested if the groups have statistically significant difference in drug responses using ANOVA.

Specifically, for each module identified using GDSC dataset for a given drug, we tested the predictive power of the module with CTRP dataset. We grouped the cell lines into three groups: (1) cell lines with alterations in *decreased sensitivity genes* only, (2) cell lines with *increased sensitivity genes* only, and (3) cell lines with *no alterations* in any of the selected genes. Subsequent ANOVA tests evaluated the statistical significance of differences in drug response among three groups of cell lines (see Section S1.3.2 for details). Since the test requires selected modules and their mutation status associated with either direction of sensitivity, we examined this measure only for NETPHIX and UNCOVER. ProGENI only ranks genes instead of identifying modules, and the directions of association (increased or decreased) are not specified.

For NETPHIX, 164 modules for 65 drugs were tested for the response with CTRP dataset (many drugs have multiple associated modules). We found that 102 modules have a statistically significant difference (p<0.05 [ANOVA], FDR <9% [BH]) and 45 drugs have at least one significant module. On the other hand, only 29 of 44 UNCOVER modules had significant difference in the response (UNCOVER identifies at most one increased and decreased module, respectively, for a drug). In summary, the results confirm that NETPHIX can identify more gene modules that are predictive of drug responses.

### Impact of NETPHIX Design Choices on the Results

Although the benefits of using protein interaction network for the identification of cancer drivers are generally accepted, it was not well investigated before how much gain the network usage provides in the context of drug responses. Here we use ANOVA tests to compare the performance of our algorithm with and without network information. Similarly, we also investigated the impact of using penalty that reinforces mutual exclusivity and different connectivity models by measuring the difference in performance in terms of the number of instances validated with ANOVA test as described in the previous section.

#### Network Information Helps Identify Drug Sensitivity Modules

NETPHIX finds a set of connected genes that are associated with drug response. To investigate the effects of using network connectivity on the performance, we ran the algorithm without connectivity constraints and compared the solutions with NETPHIX modules.

NETPHIX finds more significant modules when network information is used (476 modules compared with 274 modules without network). The number of drugs with at least one associated module is also larger (194 drugs with network versus 175 drugs without network). As shown in Figure S2A, without network only 57 of 88 tested modules were confirmed, whereas 102 of 164 tested modules were confirmed when connectivity was imposed. In addition, only 40 drugs had at least one confirmed module without network compared with 45 drugs with network. Overall, the results show that network information helps find more modules that are predictive of drug sensitivity.

Imposing penalty on overlapping mutations may improve drug sensitivity module identification.

Our objective function in ILP has an option to include penalties to further penalize overlapping mutations and enforce mutual exclusivity between mutations. We investigated the effects of using penalties on the performance by running the algorithm *without penalties* in the objective function and compared with our results obtained when penalties are used (Figure S2B). We observe that NETPHIX finds a lesser number of significant modules when penalties are not included (460 modules compared with 476 modules in the original solution), although the numbers of drugs with at least one associated modules are similar (192 drugs without penalty versus 194 drugs with penalty). In terms of the number of drugs/modules confirmed in CTRP dataset with ANOVA, 88 modules of 152 tested modules were confirmed without penalty, whereas 102 modules of 164 tested modules were confirmed with penalty. In addition, 41 drugs had at least one confirmed module without penalty compared with 45 drugs with penalty.

#### Combined versus Separate Connectivity Model

Next, we compared the performance of two connectivity models, the combined model where all selected genes are connected and the separate model in which two modules are identified for increased and decreased sensitivity separately (Figure S2C). It was not immediately obvious which approach would be more successful. On the one hand, one can hypothesize that genes responsible for either type of response are functionally related. However, it was also possible that mutations associated with drug resistance may occur in a separate module such as genes related to drug metabolism. A drug may have multiple drug targets, which may lead to separate modules.

We found that a similar number of modules were identified with the combined model and the separate model (286 versus 278 modules) for 177 versus 170 drugs. However, the combined connectivity model has a slightly higher percentage of confirmed modules/drugs. Ninety-eight modules were tested with CTRP dataset for both combined and separate models, and 65 and 61 modules were confirmed, respectively. In terms of the number of drugs with at least one confirmed module, the combined model has 37 of 57 drugs confirmed, whereas the separate model has the same number of drugs confirmed out of 59.

#### Network Properties of NETPHIX Modules

NETPHIX is designed to choose modules in which genes are highly connected, which helps identify drug sensitivity modules as discussed above. The selected modules are relatively small (the average size of 3.86) and densely connected (the average edge density of 0.55). Since NETPHIX modules are densely connected, the genes in the selected modules are naturally close in terms of distance in the network (the mean of average distances =1.78, Figure S2D). The proximity in the network means that the genes are more likely to be functionally related. In addition, the genes with the same direction of association (decreased or increased) tend to be closer to each other than the genes associated in opposite direction, although the two groups of genes are still close in the network (the mean of average distances between the two groups =1.96).

### Case Studies: NETPHIX Identifies Mutated Subnetworks Associated with Drug Responses

Many of the modules identified by NETPHIX are putative drug biomarkers supported by previous studies and provide interesting further insights related to drug action. We analyzed the identified modules associated with a few drugs in more detail.

#### Drugs Targeting RAS/MAPK Pathways

RAS/MAPK pathway regulates growth, proliferation, and apoptosis and is often dysregulated in various cancers (Figure 3G). Among the most common mutations of this pathway are mutations of BRAF/KRAS/NRAS. Interestingly, all modules associated with increased sensitivity to MEK inhibitors (Selumetinib, Trametinib, CI-1040, PD0325901, Refametinib) and an ERK inhibitor (VX-11e) included BRAF and KRAS mutations. NRAS mutations were included in 12 of 20 such modules. All these six drugs act by blocking MEK1/MEK2 or ERK genes that are immediately downstream of BRAF/KRAS/NRAS and the increased sensitivity attributed to the alterations in this subnetwork is consistent with the action of these drugs. Modules associated with decreased sensitivity to the drugs are more diverse but NETPHIX frequently selected the module of ERBB2 amplification, MYC and RB1 mutations (four times) or the module with TP53 mutations (eight times). All the genes in the modules are related to the MAPK/ERK signaling pathway. The mutation status of BRAF and KRAS, the core members of the pathway, was previously identified as predictors of MEK inhibitors, although KRAS mutations can affect drug responses differently depending on the mutation types (Nakayama et al., 2008; Li et al., 2018; Sun et al., 2014). ERBB2 is a receptor protein that signals through this pathway, whereas MYC, RB1, and TP53 are downstream of the MAPK/ERK signaling pathway. RB1 was found to be associated to the resistance to MEK inhibitors Gong et al. (2019) and MYC degradation by inhibition of MEK leads to an increase in both ERBB2 and ERBB3 mRNA expression, causing intrinsic drug resistance Sun et al. (2014). TP53 mutations are associated with multiple drug resistance (Keshelava et al., 2001; Najem et al., 2017). These findings indicate that the alterations in different components of the same pathway can contribute to drug sensitivity in different ways.

In contrast to the response to MEK1/2 and ERK2 inhibitors, the drugs directly targeting BRAF are associated with more heterogeneous modules. Although all BRAF inhibitors (except HG6-64-1) commonly exhibit increased sensitivity in BRAF mutant cell lines, KRAS mutations help the action of type II BRAF inhibitors (AZ628) but develop resistance to type I inhibitors such as Dabrafenib, PLX-4720, and SB590885, which is consistent with the previous findings Sanchez-Laorden et al. (2014). This suggests that patient-specific mutational profiles can provide important clues in predicting drug response.

#### Drugs Targeting Histone Deacetylases

Histone deacetylase (HDAC) inhibitors are cytotoxic drugs, used to destroy cancer cells by inhibiting cell division and causing cell death. HDAC is frequently dysregulated in cancer, and although HDAC inhibitors, targeting HDAC proteins, have a wide range of effects including cell-cycle arrest and apoptosis, the mechanisms of drug action are still uncertain. NETPHIX identified several modules associated with HDAC inhibitor sensitivity. For example, Vorinostat is a pan-HDAC inhibitor and inhibits class I and class II HDAC enzymes. NETPHIX found that the drug response to Vorinostat is associated with CREBBP mutation for increased sensitivity and SMAD4 deletion and DACH1 mutation for decreased sensitivity (Figure S3A). Several studies found that cell lines with CREBBP mutations are sensitive to Vorinostat treatment (Mullighan et al., 2011; Andersen et al., 2012). SMAD4 inactivation is implicated in tumor malignancy and resistance to multiple drugs (Papageorgis et al., 2011; Chen et al., 2014).

On other hand, Belinostat and CUDC-101 are found to be associated with CDKN2D deletion (decreased sensitivity), whereas TP53 deletion and MAPK7, MYC mutations are associated with increased sensitivity of the drug. Consistent with our findings, studies found that HDACis were preferentially cytotoxic to cells with mutant TP53 (Blagosklonny et al., 2005) but CDKN2A deletion is linked to poor effectiveness in combination therapy of HDACi with another agent (Chen et al., 2010).

Drug resistance is often observed for HDAC inhibitors, and combined treatments of HDACi with other anticancer drugs have demonstrated promising effects in clinical studies (Suraweera et al., 2018). As discussed below, based on NETPHIX modules we identified multiple candidates of combination therapy with HDACi that are also supported by previous evidences.

### NETPHIX Modules Suggest Candidates for Drug Combination Therapy

**Figure 4:**
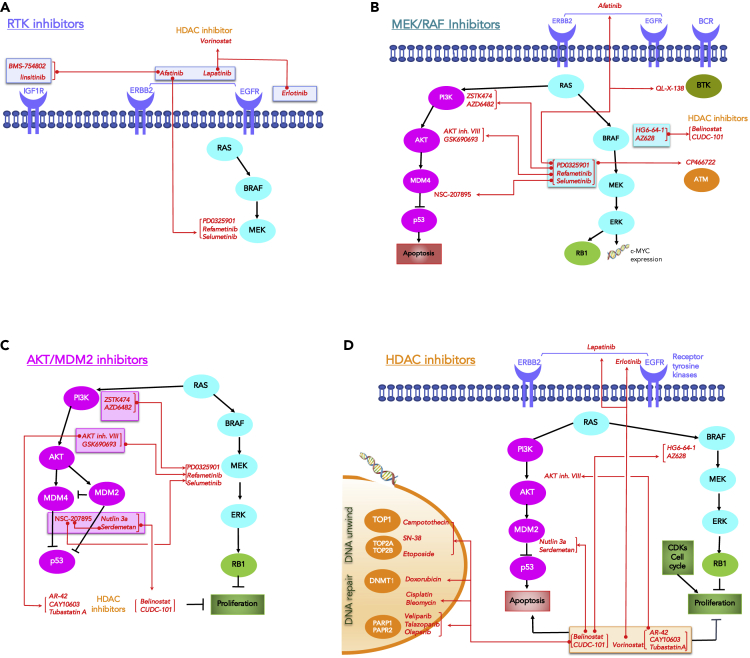
Examples of Predicted Drug Combinations Supported by Independent Evidence The candidate drug pairs (connected by red lines) that are identified based on NETPHIX modules and supported by the literature survey are shown. (A–D) The panels are organized by different drug target pathways: (A) RTK inhibitors (inside blue boxes); (B) MEK/RAF inhibitors (turquoise boxes); (C) AKT/MDM2 inhibitors (magenta boxes); (D) HDAC inhibitors (orange boxes). The name of each drug is displayed next to its target protein, and their predicted combination partners are connected by red lines. Drugs with the same interaction partners are connected by brackets. The genes/nodes are color coded as follows: orange (DNA processing), magenta (PIK/AKT/mTOR pathway), turquoise (MAPK pathway), green (cell cycle), blue half-moons (Receptors Tyrosine Kinases), dark green (BRT is a non-receptor Tyrosine Kinase).

We hypothesize that pairs of drugs can potentially have synergistic effects if they are associated with similar modules but the genes in the modules are associated with opposite directions of drug responses. By analyzing the modules for pairs of drugs with such property, we identified 153 drug pairs (Table S3). Although the systematic validation could not be performed owing to the lack of validation dataset, we found evidences in the literature for the efficacy of many predicted drug combinations. In Figure 4, we show examples of the identified combination candidates of drugs for which we found supporting evidences from the literature. The drugs are categorized by the target pathways. For example, Afatinib, a receptor tyrosine kinase inhibitor, has associated modules of KRAS, NRAS (mutations) for decreased sensitivity and EGFR, ERBB2 (amplification), and ARAF (deletion) for increased sensitivity. This suggests that it might be beneficial to use Afatinib in combination with MEK 1/2 and ERK2 targeting drugs discussed above (Figures 3C–3F). Indeed, studies showed that Afatinib and Selumetinib work synergistically (Sun et al., 2014) and clinical trials for combination therapy are currently underway (https://clinicaltrials.gov/ct2/show/NCT02450656). In addition to Selumetinib, other MEK inhibitors, such as Refametinib and PD0325901, have associated modules similar to Afatinib but in opposite direction, and the efficacy of PD0325901 and Afatinib combination is also reported (Lin et al., 2019). Indeed, drugs targeting the same molecules are typically predicted to have similar drug combination partners (Figure 4).

HDAC inhibitors are found to improve drug efficacy when combined with other anti-cancer drugs (Figure 4D). An example is the combination of Vorinostat and Lapatinib (Figure S3A and S3B). Lapatinib is a drug that inhibits EGFR/ERBB2, and Vorinostat is a histone deacetylase (HDAC) inhibitor. Lapatinib in general enhances the antitumor activity of the histone deacetylase inhibitor synergistically (LaBonte et al., 2011). Vorinostat has been shown to improve how well Lapatinib kills cancer cells in clinical trials (https://clinicaltrials.gov/ct2/show/NCT01118975) and can also improve the therapeutic efficacy of other RTK inhibitors such as Erlotinib (Leone et al., 2015).

## Discussion

We developed a new computational method, NETPHIX (NETwork-to-PHenotype assocIation with eXclusivity), for the identification of mutated subnetworks that are associated with a continuous phenotype. Using simulations and analyzing large-scale drug screening datasets, we showed that NETPHIX can uncover the subnetworks associated with response to cancer drugs. We found many statistically significant and biologically relevant modules associated with drug response, including MAPK/ERK signaling-related modules associated with opposite response to drugs targeting RAF, MEK, and ERK genes. The genetic alteration status in many of the identified modules indeed make differences in cell survival rates, as validated with an independent dataset. Overall, the modules identified by NETPHIX are in good correspondence with the action of the respective drugs, suggesting that NETPHIX can correctly identify relevant modules and the modules can thus be used to predict potential patient-specific drug combinations and to provide guidance to personalized treatment.

The mutual exclusivity property has been shown to be helpful for identifying cancer drivers, but the hypothesis that the property can also help define mutated subnetworks associated with drug response was not well investigated before. We demonstrate that the preferential selection of mutually exclusive genes can improve the performance of the method, although the improvement is moderate. We note that the set cover approach itself might favor the genes that are mutually exclusive to maximize the coverage with the smallest number of genes. We hypothesize that the improvement is related to the fact that mutual exclusivity increases the probability that selected genes are cancer related. However, further studies may be necessary regarding the relationship between mutually exclusive mutations and drug sensitivity. By removing the mutual exclusivity condition, NETPHIX can also be used for the more general problem of linking continuous phenotypes (not specific to cancer) with genetic alterations.

Interestingly, although one might assume that genes affecting drug resistance are not necessarily functionally related to the genes increasing drug sensitivity, we found that the combined connectivity model slightly outperforms the separate connectivity model, indicating that the two groups of genes in fact might be related. We also found that, even for the separate model, the sensitivity modules are relatively close to each other (Figure S2D), which suggests that the genes whose alterations are associated with the same drug response may belong to the same or related pathways.

Although the combined connectivity model works better in terms of the number of significant and validated modules, there are a few drugs for which the separate model provides different insights on drug action. For example, for Cytarabine, both the combined and the separate model identified statistically significant modules, which are also confirmed in CTRP dataset. In particular, the module associated with Cytarabine in the separate model (Figure S3C) includes UGT2B17 and CYP2E1 deletion associated with decreased sensitivity. Both enzymes are hypothesized to be important players in the metabolism of common drugs (Guillemette et al., 2014; Garcia-Suastegui et al., 2017). Since drug metabolism pathways are typically not part of cell growth-related cancer-driving pathways, the separate connectivity approach can provide insights that the combined model cannot provide in such cases.

The applicability of NETPHIX can go far beyond the drug response discussed in this paper, to any continuous cancer phenotypes. A simplified version of our method was used to investigate the genetic aberrations associated with mutational signatures, by which the mutagenic processes underlying the cancer genomes were elucidated (Kim et al., 2020). In that case, the phenotype of interest was the strength of a mutagenic process as measured by the mutation counts of the corresponding mutational signature. We expect that NETPHIX will find broad applications in many other types of network-to-phenotype association studies, especially in the context of cancer.

### Limitations of the Study

NETPHIX is focused on the identification of mutated subnetworks associated with drug response. Such mutated modules might not always exist as, for example, some drugs are designed to reduce expression of overexpressed genes. In addition, there is no guarantee that the association identified by NETPHIX is causal, although the proximity of identified subnetworks to the drug targets suggests that causal relations are quite likely. Finally, in case of systematic co-occurrence of alterations (for example, when two genes are co-amplified) NETPHIX might report only one of these genes owing to the nature of set cover-based algorithms.

### Resource Availability

#### Lead Contact

Further information and requests for resources and information should be directed to and will be fulfilled by the Lead Contact, Teresa M. Przytycka (przytyck@ncbi.nlm.nih.gov).

#### Materials Availability

This study did not generate new unique reagents.

#### Data and Code Availability

The source code and the datasets used for and generated during this study are available at https://www.ncbi.nlm.nih.gov/CBBresearch/Przytycka/index.cgi#netphix.

## Methods

All methods can be found in the accompanying Transparent Methods supplemental file.

## Supplemental Information

Document S1. Transparent Methods and Figures S1–S4

Table S1. NETPHIX Modules For each drug in the GDSC dataset, final modules are selected satisfying the following constraints: (1) has the best p value (<0.05) among all modules associated with the drug; (2) is a maximal set (is not a subset of another).

Table S2. Validation with CTRP Dataset NETPHIX modules identified with GDSC are validated with CTRP dataset using ANOVA test.

Table S3. Drug Combination Candidates Pairs of drug modules with opposite direction of associations are selected as potential drug combinations. We also added the supporting evidence from the literature.
