# Supplementary material for: Identifying Drug Sensitivity Subnetworks with NETPHIX
Source: iScience. 2020 Sep 29;23(10):101619. doi: 10.1016/j.isci.2020.101619 (PMC7566085; doi:10.1016/j.isci.2020.101619)
Supplement: Document S1. Transparent Methods and Figures S1–S4 [file mmc1.pdf]

iScience, Volume *23*

Supplemental Information

Identifying Drug Sensitivity Subnetworks with NETPHIX

Yoo-Ah Kim, Rebecca Sarto Basso, Damian Wojtowicz, Amanda S. Liu, Dorit S. Hochbaum, Fabio Vandin, and Teresa M. Przytycka

Supplemental Information

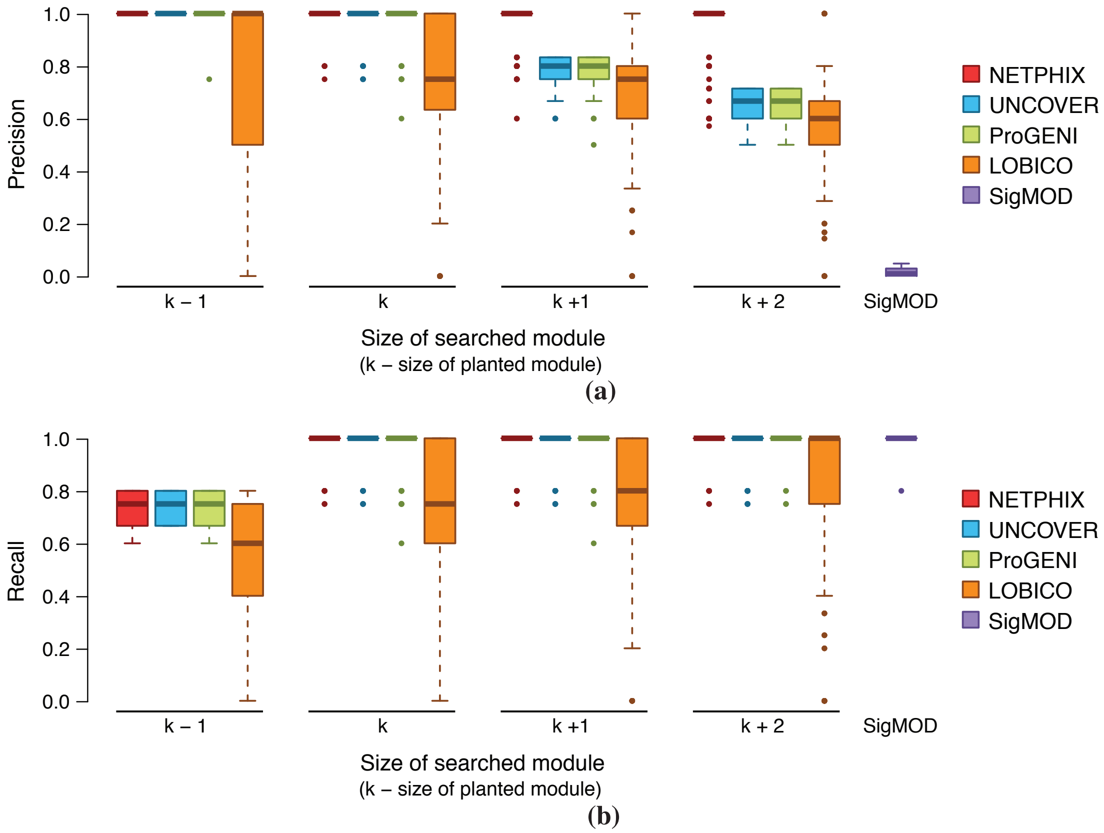

Figure S1: Method comparison on simulated data (Related to Figure 2). **(a)** Precision and **(b)** Recall for the modules identified by NETPHIX (red), UNCOVER (blue), ProGENI (green), LOBICO (orange), and SigMOD (purple).

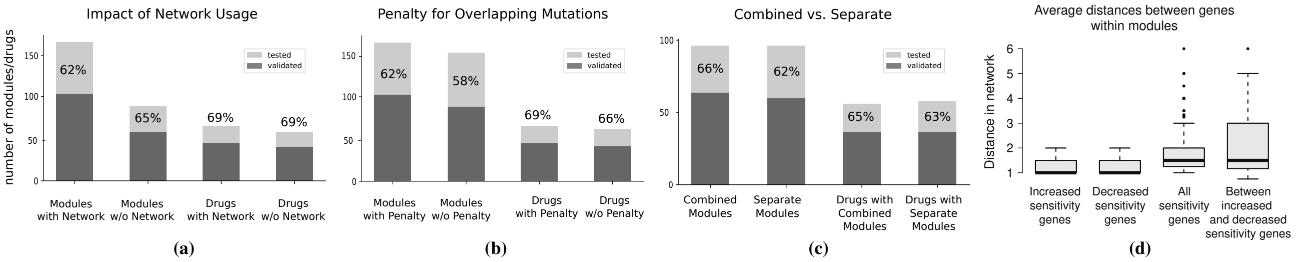

Figure S2: Impact of design choices on the performance of the algorithm (Related to Figure 3) (a) Comparison between runs with and without network information. The number of tested modules/drugs with CTRP (tested) and the number of confirmed modules (validated) with ANOVA test ($p < 0.05$) are shown. Drugs are counted when there is at least one associated modules that are validated (b) Comparison between runs with and without penalty promoting mutual exclusivity (c) Comparison between the combined and separate connectivity models. (d) Average distances between genes in the selected modules. Distances for genes associated with increased sensitivity, decreased sensitivity, all sensitivity, and between decreased and increased sensitivity genes are shown.

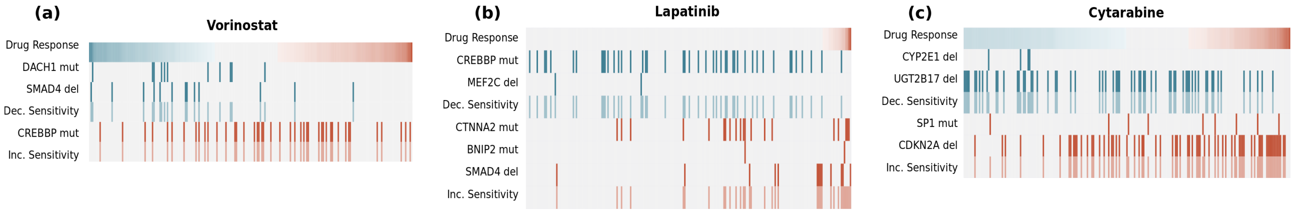

Figure S3: Modules identified by NETPHIX (Related to Figure 3). (a-b) Sensitivity module for Vorinostat (a) and Lapatinib (b). The two modules associated with the drugs are similar but they are associated with opposite directions. The efficacy of combination therapy with Lapatinib and Vorinostat is confirmed in clinical trials. (c) Sensitivity module for Cytarabine identified based on the separate connectivity model.

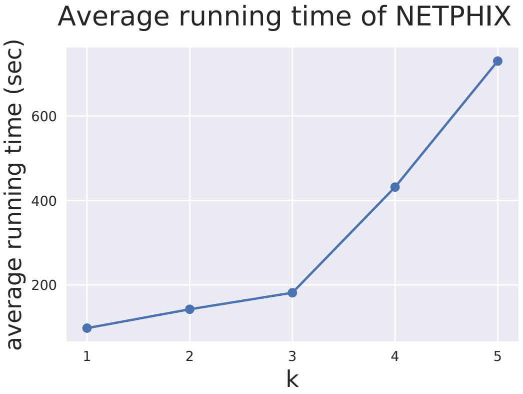

Figure S4: The average running times of NETPHIX over different k's (Related to Section S1.1.3)

S1 Transparent Methods

S1.1 NETPHIX method

S1.1.1 Formal definition of the computational problem for NETPHIX

We are given a graph $G = (V, E)$, with vertices $V = \{1, \ldots, n\}$ representing genes and edges E representing interactions among genes. Let P denote the set of m patients (or cell lines). For each sample $j \in P$, we are also given a phenotype profile value $w_j \in \mathbb{R}$ which quantitatively measures a phenotype (e.g., drug response in our study). Let $P_i \subseteq P$ be the set of patients in which gene $i \in V$ is altered. We say that a patient $j \in P$ is *covered* by gene $i \in V$ if $j \in P_i$ i.e. if gene i is altered in sample j. We say that a sample $j \in P$ is *covered* by a subset of genes (or vertices) $S \subseteq V$, if there exists at least one vertex v in S such that $j \in P_v$.

For simplicity of description, we start with the formulation in the case where the association is in one direction, for example, with increased drug sensitivity. Later we will show how to extend the problem to accommodate the case where mixed associations are allowed in the same module. Our goal is to identify a connected subgraph S of G of at most k vertices such that the sum of the weights of the samples covered by S is maximized. The weights are computed based on drug sensitivity. To identify functionally complementary mutations, we can penalize coverage overlap when a sample is covered more than once by S by assigning a penalty p_j for each of the additional times sample j is covered by S. Let $c_S(j)$ be the number of times element $j \in P$ is covered by S. For a set S of genes, we define its weight $W(S)$ as:

$$W(S) = \sum_{j \in \cup_{s \in S} P_s} w_j - \sum_{j \in \cup_{s \in S} P_s} (c_S(j) - 1) p_j \tag{1}$$

Thus, we define the optimization problem for one-side association as follows: Given a graph G defined on a set of n vertices V, a set P, a family of subsets $P = \{P_1, \ldots, P_n\}$ where for each i, $P_i \subseteq P$ is associated with $i \in V$, weights w_j and penalties $p_j \geq 0$ for each sample $j \in P$, find the subset $S \subseteq V$ of $\leq k$ connected vertices maximizing $W(S)$.

Since genetic alterations may affect the increase or decrease of drug sensitivity, we extend the

problem to identify genes with associations in both directions in one module. Considering genes with increased and decreased sensitivity simultaneously can pick up stronger signals of associations and allow to take into account the interactions between alterations affecting drug responses in different ways. Let I include the genes associated with increased sensitivity overall (i.e., genes i with positive total weights, $\sum_{j\in P_i} w_j \geq 0$) and D is the set of genes associated with decreased sensitivity overall (i.e., genes i with negative total weights, $\sum_{j\in P_i} w_j < 0$). Our objective function is then defined as follows:

$$W(S) = \sum_{j\in\cup_{s\in S\bigcap I}P_s} w_j^I - \sum_{j\in\cup_{s\in S\bigcap I}P_s} (c_{S\bigcap I}(j)-1)p_j^I + (\sum_{j\in\cup_{s\in S\bigcap D}P_s} w_j^D - \sum_{j\in\cup_{s\in S\bigcap D}P_s} (c_{S\bigcap D}(j)-1)p_j^D) \quad (2)$$

where we define $w_j^I = w_j$ and $w_j^D = -w_j$. We considered two versions of connectivity constraints among the associated genes as illustrated in Figure 1b. In the first model, we insisted that all selected genes should be connected whether they are associated with increased or decreased sensitivity. In the second model, we ensured the connectivity of genes with the same direction of association, resulting in two connected components in a solution (one for increased and the other for decreased sensitivity).

Although the problem is NP-hard (by a reduction to set cover) even for the simple one-sided case without network constraints, we formulated it as an integer linear program as described in the next subsection, which can be solved using a optimization software package such as CPLEX.

S1.1.2 ILP formulation of NETPHIX

Let x_i be a binary variable (denoted with $x_i \in \mathbb{B}$) equal to 1 if gene $i \in V$ is selected and $x_i = 0$ otherwise. Let z_j^I(resp., z_j^D) be a binary variable equal to 1 if sample j is covered by a gene $i \in I$ (resp., $i \in D$) and 0 otherwise. Let $y_j^I (resp., y_j^D)$ denote the number of genes in I (resp., D) cover sample j in the solution. Finally, let w_j be the weight of sample j and p_j be the penalty for sample j. When sample j is covered by a gene in I, the weight and penalty remain the same $w_j^I = w_j$. When j is covered by a gene in D, $w_j^D = -w_j$. Our ILP formulation for the combined model is defined as follows:

$$z(q) = \max \quad \sum_j (w_j^I + p_j^I) z_j^I - \sum_j p_j^I y_j^I + \sum_j (w_j^D + p_j^D) z_j^D - \sum_j p_j^D y_j^D \tag{3}$$

$$\text{s.t.} \sum_i x_i \leq k, \tag{4}$$

$$y_j^I = \sum_{i:j \in P_i, i \in I} x_i, \qquad \forall j \tag{5}$$

$$y_j^D = \sum_{i:j \in P_i, i \in D} x_i, \qquad \forall j \tag{6}$$

$$y_j^I \geq z_j^I, \qquad \forall j \tag{7}$$

$$y_j^D \geq z_j^D, \qquad \forall j \tag{8}$$

$$z_j^I \geq y_j^I / k, \qquad \forall j \tag{9}$$

$$z_j^D \geq y_j^D / k, \qquad \forall j \tag{10}$$

$$x_i, z_j \in \mathbb{B}, y_j \in \mathbb{D} \qquad \forall i, j \tag{11}$$

$$\sum_{l:il \in E} x_l \geq C(k-1)(x_i - 1) + C\left(\sum_{l \in V} x_l - 1\right) \qquad \forall i \in V \tag{12}$$

Constraint (4) impose that the total number of sets (i.e., selected genes) in the solution is at most k. Constraints (5) and (6) define how many times each sample has been covered by genes in I and D, respectively. Constraints (7) (resp., Constraints (8)) ensure that for each sample $j \in P$, if j is covered by increased (resp., decreased) sensitivity genes in the current solution then the number of times j is covered by I (resp., D) in the solution is at least 1. Constraints (9) (resp., Constraints (10)) impose that for each element (sample) $j \in P$, if j is covered by at least one increased (resp., decreased) sensitivity gene in the current solution then j is covered by I (resp., D).

Constraints (12) were used to ensure the high connectivity of a selected module (the combined connectivity model). Specifically, the constraints enforce that each selected gene is connected with at least C fraction of genes in the selected module (other than the gene itself). Note that if $C \geq 0.5$, the module is a connected subgraph since for any two non-adjacent vertices, they must have a common neighbor ($C = 0.5$ is used in our analysis). In our study, we used a functional interaction network

(from STRING database), which is relatively dense. For sparse networks where highly connected components are rare, we may use an alternative approach based on a branch-and-cut algorithm to ensure the connectivity [Fischetti et al., 2017, Bomersbach et al., 2016, Wang et al., 2017].

Note that Constraints (12) forces the connectivity among all selected genes regardless of the directions of association. For the separate connectivity model, we identify candidate modules so that the connectivity is only enforced among the genes in I and D, separately. In this case, we replace the connectivity constraints given in (12) with the following constraints.

$$\sum_{l:il \in E, l \in I} x_l \geq C(k-1)(x_i - 1) + C\left(\sum_{l \in I} x_l - 1\right) \quad \forall i \in I \tag{13}$$

$$\sum_{l:il \in E, l \in D} x_l \geq C(k-1)(x_i - 1) + C\left(\sum_{l \in D} x_l - 1\right) \quad \forall i \in D \tag{14}$$

S1.1.3 Parameters

To obtain a pool of candidate modules for each drug, we generated ILP instances with different sizes k ($k = 1$ to 5) and two connectivity options (the combined and separate model). The objective function can include a penalty to reinforce mutual exclusivity. As for the penalty for increased sensitivity p_j^I, we use the average of the positive phenotype values if the original value of the element was positive $(w_j > 0)$ and assign a penalty equal to its absolute value otherwise. The penalty for decreased sensitivity p_j^D is computed in the opposite way. The negative of the average of the negative phenotype values is used if the original value of the element was negative $(w_j < 0)$ and assign a penalty equal to its absolute value otherwise. The penalties are set to be zero when no penalty is imposed.

We solved the ILP instances to optimality using CPLEX, which can be run in a reasonable amount of time (See Figure S4 for running times for the simulation instances with different k's). For the instances requiring a large amount of resources solving ILP, we set the time limit of 24h and the memory space limit of 10 GB.

S1.1.4 Selecting final modules

For each candidate module, we run a permutation test to assess the statistical significance of association and select maximal modules among significantly associated ones. Note that we allow to choose multiple modules associated with a drug in the final solution because it is possible that multiple functional components are associated with drug response.

Permutation test: For each candidate module, we assess the statistical significance of the association between their alteration profile and drug response by a phenotype permutation test. In the phenotype permutation, the dependencies among alterations in genes are maintained, while the association between alterations and the phenotype is removed. Specifically, a permuted dataset under the null distribution is obtained as follows: the graph $G = (V, E)$ and the sets $P_i, i \in V$ are the same as observed in the data; the values of the phenotype are randomly permuted across the samples (Figure 1c). Once we find the optimal solution for the original instance, we can run ILP as a feasibility test simply checking if a permuted instance has a solution with the objective value that is greater than or equal to the optimal.

To estimate the p-value for the solutions obtained by ILP, we used the following standard procedure: 1) we run an algorithm on the real data $\mathcal{D}$, obtaining a solution with objective function $o_{\mathcal{D}}$; 2) we generate N permuted datasets as described above; 3) we run a feasibility test by simply checking if a permuted instance has a solution with the objective value greater than or equal to $o_{\mathcal{D}}$; 4) the p-value is then given by $(e + 1)/(N + 1)$, where e is the number of permuted datasets in which our algorithm found a solution with objective function $\geq o_{\mathcal{D}}$. We used $N = 100$ permutations in our analysis and let p_{best} is the most significant p-value for the drug among different parameters. We only considered modules with p-value = p_{best} . If $p_{best} < 0.05$ (FDR $< 10\%$, BH), we considered those modules as significantly associated modules.

Selecting maximal modules: Among all significantly associated modules obtained based on the permutation test, we remove redundant modules by selecting only maximal modules. In other words, let $M_1, M_2, ..., M_t$ be the set of significantly associated modules for a drug. For any two modules M_i and M_j such that $M_i \subset M_j$, we only include M_j in the final solution for the drug. Therefore, for two

overlapping modules, when one is not a proper subset of the other, both modules may be included.

S1.2 Datasets

Drug sensitivity dataset: The Genomics of Drug Sensitivity in Cancer Project (`https://www.cancerrxgene.org/`) consists of drug sensitivity data generated from high-throughput screening using fluorescence-based cell viability assays following 72 hours of drug treatment. In particular, we considered the area under the curve for each experiment as a phenotype. These scores are provided in the file `portal-GDSC_AUC-201806-21.txt` available through the DepMap data portal (`https://depmap.org`) for 265 compounds and 743 cell lines, with 736 having alteration data available through the DepMap portal. For the DepMap experiments [Stransky et al., 2015, Barretina et al., 2012], we used the alteration provided at `https://depmap.org/portal/download/all/`. We downloaded the data on July 6th 2018. In particular we used mutation data from the file `portal-mutation-201806-21.csv` that includes binary entries for 18,652 gene-level mutations. Additionally, we considered 22,746 amplifications and 22,746 deletions computed from the gene copy number data in `portal-copy_number_relative-2018-06-21.csv`, with an amplification defined by a copy number above 2 and a deletion defined by a copy number below -1. Removing genes not present in the interaction network (see below for the details of interaction network data), we collected 26,917 gene-level alteration profiles (combining amplification, deletion and mutation).

We also utilized an independent drug response dataset from the Cancer Therapeutics Response Portal (CTRP) for validation [Seashore-Ludlow et al., 2015]. The drug screening results were downloaded from `https://portals.broadinstitute.org/ctrp/`(Version 2). The area under the curve (AUC) values in `v20.data.curves_post_qc.txt` were used for drug response phenotypes (from `CTRPv2.0_2015_ctd2_ExpandedDataset.zip` file downloaded on August 2nd, 2019).

Preprocessing drug sensitivity data: For every drug response profile, we excluded samples with missing values for that phenotype, which results in a different number of samples for each phenotype. The number of samples varied between 240 and 705. To generate drug sensitivity values for

the patients, we took the negatives of cell viability (i.e., increased cell survival indicates decreased sensitivity to the drug and vice versa) and then normalized the phenotype values before running the algorithm, by using standard z-scores (subtracting the average value $\sum_{j \in J} w_j/m$ from each weight w_j and dividing the result by the standard deviation of the (original) w_j's), in order to have both positive and negative phenotype values. We excluded genes with low mutation frequency (present in less than 1% samples) from our analyses.

Interaction network: For functional interactions among genes, we used the data downloaded from STRING database version 10.0 (`https://string-db.org`). The data integrates multiple types of interactions including physical interactions. We only included the edges with high confidence scores ($\geq$ 900 out of 1000) as an input to NETPHIX. The resulting interaction network includes 9,215 nodes and 160,249 edges.

S1.3 Evaluation Details

S1.3.1 Running simulated experiments

For the background of simulation data, we use the same gene alteration table and interactions from drug sensitivity dataset described previously in Section S1.2. The phenotype values for individual samples are randomly drawn from normal distribution $N(0, 1)$. We then planted randomly generated phenotypes and associated modules to the background as follows.

Phenotypes: α fraction of patients $P(\alpha)$ ($\alpha = 0.1, 0.2$, and 0.3) were randomly selected and assigned phenotype values drawn randomly from $N(z, 0.5)$ where z is a z-score corresponding to a cumulative p-value p ($p =$ 0.005, 0.1, 0.99, and 0.995).

Associated gene modules: we randomly selected a gene set $S(k)$ of size k ($k = 3, 4$, and 5) and added random alterations in $S(k)$ for patients $P(\alpha)$ so that each patient in $P(\alpha)$ has an alteration in exactly one gene in $S(k)$. Therefore, the added alterations among the patients $P(\alpha)$ are mutually exclusive although there may be overlapping mutations due to the background alterations. We also added random edges among the genes $S(k)$ so that they satisfy the density constraints ($C = 0.5$)

We generated 10 random instances for each combination of parameters (k, α, z) and ran the module identification algorithms.

For LOBICO [Knijnenburg et al., 2016], we used its R implementation (`https://github.com/clareli9/rlobico`,release 2018/7/27) with the default parameter settings, except the logic function parameters (K and M) and the maximum running time. The OR logic model with $K = k$ and $M = 1$ was used for increased sensitivity modules and the AND logic module with $K = 1$ and $M = k$ for decreased sensitivity modules, where k is the size of the searched module. We limited the running time of ILP instances to be 24h and reported the best current solution (which may be suboptimal) when the program stops.

For ProGENI, we downloaded the program from github (`https://github.com/KnowEnG/ProGENI`, release 2017/7/12). ProGENI originally utilized gene expression information for drug prediction but for comparison with NETPHIX, we used gene alteration profiles instead. The profiles for genes are computed by summing over mutation, deletion and amplification for each gene. Therefore, each entry in the matrix can have a value between 0 and 2 as deletions and amplifications cannot co-occur. We ran a robust version of ProGENI, in which gene prioritization is performed on randomly selected 80% of samples 50 times repeatedly and the resulting ranked lists are aggregated to produce the final ranking of genes.

S1.3.2 Method comparison with real drug screening dataset.

Identifying modules using different methods. UNCOVER modules are obtained by setting $k = 3$ (module size) as presented in [Sarto Basso et al., 2019] and for decreased and increased sensitivity separately. The significance of each module is assessed using permutation tests (using 100 permuted instances), and we consider the modules with $p < 0.05$ as significant modules and used for further analysis. The rankings of genes in ProGENI are computed by performing RobustProGENI with bootstrap sampling rate of 95% and 100 runs.

Computing distance information. To compute the distances from drug targets to the selected modules by the algorithms, we used the drug target information given in Table S1. For each drug, we only used the drug targets present in the functional network that are reachable from the selected modules and computed the average distance for all pairs of genes. The identified modules are used for NETPHIX and UNCOVER while 5 and 20 top-ranked genes are used for ProGENI. We also included 5

and 20 randomly selected genes for control.

For NETPHIX modules, we also computed the average distances within modules. The distances are computed by computing the pairwise shortest distances within modules and take the average distances.

Response prediction using random forest regression. We ran RandomForestRegressor in scikit-learn package to learn and test the models. First, we ran 3-fold nested cross validation with GDSC dataset for each drug. For each of training sets, the best model is learned based on 3-fold cross validations inside the training set, with the best parameters estimated using GridsearchCV with combinations of parameters (n_estimators = (10, 100), max_depth = (None, 10, 100), and min_samples_split = (1, 2, 3)). To measure the performance, we used probabilistic concordance index (PCI) defined as in [Costello et al., 2014, Cokelaer et al., 2015, Emad et al., 2017]. The PCI metric compares the ranks of the predicted values and actual AUC scores to compute the prediction power.

For NETPHIX, we merged all modules significantly associated with the response for each drug and used them as features for regression. The number of features for each drug vares between 3 to 15 in total. UNCOVER finds at most one significant module for decreased and increased sensitivity respectively, and genes in both directions are used as features (total of 3 to 6 genes). For ProGENI, we used 20 top ranked genes as features for regression.

We found the drug response profiles for 76 drugs in both CTRP and GDSC datasets, among which 44 drugs with consistent drug response profiles were used (pearson correlation coefficient > 0.25) for validation. For the 44 drugs, we trained the model with GDSC datasets for the modules identified by the algorithm and tested with the responses in CTRP. The hyperparameters were learned using 4-fold cross validation in GDSC among the same parameter combinations given above and the best models were used to predict the drug response values in CTRP.

Validation with CTRP dataset using ANOVA test. To test if the alteration status of selected genes are associated with different drug responses, we also performed ANOVA tests by testing UNCOVER and NETPHIX modules identified from GDSC dataset in the drug responses in CTRP. For each module, we divided the cell lines into three groups; The cell lines (C_I) with alterations in increased

sensitivity genes but no alterations in decreased sensitivity genes, the cell lines (C_D) with alterations in decreased sensitivity genes but no alterations in increased sensitivity genes, and the cell lines (C_N) with no mutations in the identified genes. We then performed ANOVA tests for the cell survival rates (AUC) in CTRP dataset for the three groups (C_I, C_D, and C_N), and the modules with $p < 0.05$ are considered as validated.

References

[Barretina et al., 2012] Barretina, J., Caponigro, G., Stransky, N., Venkatesan, K., Margolin, A. A., Kim, S., Wilson, C. J., Lehar, J., Kryukov, G. V., Sonkin, D., Reddy, A., Liu, M., Murray, L., Berger, M. F., Monahan, J. E., Morais, P., Meltzer, J., Korejwa, A., Jane-Valbuena, J., Mapa, F. A., Thibault, J., Bric-Furlong, E., Raman, P., Shipway, A., Engels, I. H., Cheng, J., Yu, G. K., Yu, J., Aspesi, P., de Silva, M., Jagtap, K., Jones, M. D., Wang, L., Hatton, C., Palescandolo, E., Gupta, S., Mahan, S., Sougnez, C., Onofrio, R. C., Liefeld, T., MacConaill, L., Winckler, W., Reich, M., Li, N., Mesirov, J. P., Gabriel, S. B., Getz, G., Ardlie, K., Chan, V., Myer, V. E., Weber, B. L., Porter, J., Warmuth, M., Finan, P., Harris, J. L., Meyerson, M., Golub, T. R., Morrissey, M. P., Sellers, W. R., Schlegel, R., and Garraway, L. A. (2012). The Cancer Cell Line Encyclopedia enables predictive modelling of anticancer drug sensitivity. *Nature*, 483(7391):603–607.

[Bomersbach et al., 2016] Bomersbach, A., Chiarandini, M., and Vandin, F. (2016). An efficient branch and cut algorithm to find frequently mutated subnetworks in cancer. In *Algorithms in Bioinformatics - 16th International Workshop, WABI 2016, Aarhus, Denmark, August 22-24, 2016. Proceedings*, pages 27–39.

[Cokelaer et al., 2015] Cokelaer, T., Bansal, M., Bare, C., Bilal, E., Bot, B. M., Chaibub Neto, E., Eduati, F., de la Fuente, A., G?nen, M., Hill, S. M., Hoff, B., Karr, J. R., K?ffner, R., Menden, M. P., Meyer, P., Norel, R., Pratap, A., Prill, R. J., Weirauch, M. T., Costello, J. C., Stolovitzky, G., and Saez-Rodriguez, J. (2015). Dreamtools: a python package for scoring collaborative challenges. *F1000Research*, 4:1030.

[Costello et al., 2014] Costello, J. C., Heiser, L. M., Georgii, E., G?nen, M., Menden, M. P., Wang, N. J., Bansal, M., Ammad-ud din, M., Hintsanen, P., Khan, S. A., Mpindi, J.-P., Kallioniemi, O., Honkela, A., Aittokallio, T., Wennerberg, K., Community, N. D., Collins, J. J., Gallahan, D., Singer, D., Saez-Rodriguez, J., Kaski, S., Gray, J. W., and Stolovitzky, G. (2014). A community effort to assess and improve drug sensitivity prediction algorithms. *Nature biotechnology*, 32(12):1202–12.

[Emad et al., 2017] Emad, A., Cairns, J., Kalari, K. R., Wang, L., and Sinha, S. (2017). Knowledge-guided gene prioritization reveals new insights into the mechanisms of chemoresistance. *Genome biology*, 18(1):153.

[Fischetti et al., 2017] Fischetti, M., Leitner, M., Ljubic, I., Luipersbeck, M., Monaci, M., Resch, M., Salvagnin, D., and Sinnl, M. (2017). Thinning out steiner trees: a node-based model for uniform edge costs. *Math. Program. Comput.*, 9(2):203–229.

[Knijnenburg et al., 2016] Knijnenburg, T. A., Klau, G. W., Iorio, F., Garnett, M. J., McDermott, U., Shmulevich, I., and Wessels, L. F. (2016). Logic models to predict continuous outputs based on binary inputs with an application to personalized cancer therapy. *Sci Rep*, 6:36812.

[Sarto Basso et al., 2019] Sarto Basso, R., Hochbaum, D. S., and Vandin, F. (2019). Efficient algorithms to discover alterations with complementary functional association in cancer. *PLoS Comput. Biol.*, 15(5):e1006802.

[Seashore-Ludlow et al., 2015] Seashore-Ludlow, B., Rees, M. G., Cheah, J. H., Cokol, M., Price, E. V., Coletti, M. E., Jones, V., Bodycombe, N. E., Soule, C. K., Gould, J., Alexander, B., Li, A., Montgomery, P., Wawer, M. J., Kuru, N., Kotz, J. D., Hon, C. S., Munoz, B., Liefeld, T., Dan?ik, V., Bittker, J. A., Palmer, M., Bradner, J. E., Shamji, A. F., Clemons, P. A., and Schreiber, S. L. (2015). Harnessing Connectivity in a Large-Scale Small-Molecule Sensitivity Dataset. *Cancer Discov*, 5(11):1210–1223.

[Stransky et al., 2015] Stransky, N., Ghandi, M., Kryukov, G. V., Garraway, L. A., Lehar, J., Liu, M., Sonkin, D., Kauffmann, A., Venkatesan, K., Edelman, E. J., Riester, M., Barretina, J., Caponigro, G., Schlegel, R., Sellers, W. R., Stegmeier, F., Morrissey, M., Amzallag, A., Pruteanu-Malinici, I., Haber, D. A., Ramaswamy, S., Benes, C. H., Menden, M. P., Iorio, F., Stratton, M. R., McDermott, U., Garnett, M. J., and Saez-Rodriguez, J. (2015). Pharmacogenomic agreement between two cancer cell line data sets. *Nature*, 528(7580):84–87.

[Wang et al., 2017] Wang, Y., Buchanan, A., and Butenko, S. (2017). On imposing connectivity constraints in integer programs. *Math. Program.*, 166(1-2):241–271.
